# Curcumin Inhibits LIN-28A through the Activation of miRNA-98 in the Lung Cancer Cell Line A549

**DOI:** 10.3390/molecules22060929

**Published:** 2017-06-03

**Authors:** Wei-Lun Liu, Jia-Ming Chang, Inn-Wen Chong, Yi-Li Hung, Yung-Hsiang Chen, Wen-Tsung Huang, Hsuan-Fu Kuo, Chong-Chao Hsieh, Po-Len Liu

**Affiliations:** 1 Department of Intensive Care Medicine, Chi Mei Medical Center, Liouying, Tainan 73657, Taiwan; medrpeterliu@gmail.com; 2 School of Medicine, College of Medicine, Fu Jen Catholic University, New Taipei 24205, Taiwan; b82401103@yahoo.com.tw; 3 Graduate Institute of Medical Sciences, College of Health Sciences, Chang Jung Christian University, Tainan 71101, Taiwan; 4 Department of pharmacology, Institute for Drug Evaluation Platform, Development Center for Biotechnology, New Taipei 22180, Taiwan; jiaming@dcb.org.tw; 5 Department of Respiratory Therapy, College of Medicine, Kaohsiung Medical University, Kaohsiung 80756, Taiwan; chong@kmu.edu.tw; 6 Department of Internal Medicine, Kaohsiung Medical University Hospital, Kaohsiung 80756, Taiwan; 7 Department of Pediatrics, Cathay General Hospital, Taipei 10630, Taiwan; 8 Graduate Institute of Integrated Medicine, College of Chinese Medicine, China Medical University, Taichung 40407, Taiwan; yhchen@mail.cmu.edu.tw; 9 Department of Psychology, College of Medical and Health Science, Asia University, Taichung 41354, Taiwan; 10 Division of Hemato-oncology, Department of Internal Medicine, Chi Mei Medical Center, Liouying, Tainan 73657, Taiwan; huangwentsung@mac.com; 11 Department of Internal Medicine, Kaohsiung Municipal Ta-Tung Hospital, Kaohsiung Medical University Hospital, Kaohsiung Medical University, Kaohsiung 80145, Taiwan; medsnail@hotmail.com; 12 Division of Cardiovascular Surgery, Department of Surgery, Kaohsiung Medical University Hospital, Kaohsiung 80756, Taiwan; chchhs@cc.kmu.edu.tw

**Keywords:** curcumin, LIN28A, metastasis, miR-98, lung cancer, MMP

## Abstract

Metastasis is common in lung cancer and is associated with poor clinical outcomes and increased mortality. Curcumin is a natural anti-cancer agent that inhibits the metastasis of various cancers by modulating the expression of micro (mi) RNAs such as miR-98, which acts as a tumor suppressor. This study investigated the effect of curcumin on miR-98 expression and in vitro cell line growth and invasiveness in lung cancer. Curcumin treatment enhanced the expression of miR-98 and reduced that of the miR-98 target gene *LIN28A* as well as *matrix metalloproteinase* (*MMP*) *2* and *MMP9* in vitro and in vivo. MiR-98 overexpression suppressed lung cancer cell migration and invasion by inhibiting LIN28A-induced MMP2 and MMP9 expression. Meanwhile, *LIN28A* level was downregulated by overexpression of miR-98 mimic. Induction of miR-98 by curcumin treatment suppressed MMP2 and MMP9 by targeting LIN28A. These findings provide insight into the mechanisms by which curcumin suppresses lung cancer cell line growth in vitro and in vivo and invasiveness in vitro.

## 1. Introduction

Non-small cell lung cancer (NSCLC) is the most common type of lung cancer in Taiwan, with a 5-year survival rate that is lower than that of other cancers. Early-stage NSCLC is asymptomatic until it spreads to other organs, at which point the optimal time window for surgery or chemotherapy may have closed. Tumor metastasis is one of the main causes of death from NSCLC, and its prevention is essential for improving patient outcome [1]. Many studies have found that MMPs, in particularly MMP2 and MMP9, are important molecules in cancer tissue remodeling. For example, they are able to catalyze fibrin, fibrinogen, plasminogen, elastin, types V, VII, IX, X, and IV collagen to facilitate malignant cell invasion and metastasis [2].

Micro (mi) RNAs are short, highly conserved noncoding RNAs 18–22 nucleotides in length that regulate gene expression at the post-transcriptional level and can function as oncogenes or tumor suppressor genes [3]. MiRNAs have been implicated in the metastatic progression of NSCLC [4,5]. The miRNA miR-98 is a tumor suppressor belonging to let-7/miR-98 family [6] that has been linked to metastasis in cancers such as oral squamous cell carcinoma and colon, ovarian, and lung cancer[7,8,9,10]. MiR-98 overexpression was shown to suppress tumor cell proliferation [11,12], epithelial-mesenchymal transition (EMT), chemoresistance [13], and metastasis [14] by targeting p21 (RAC1)-activated kinase 1, SNAIL, and LIN28 [15,16,17], while its inhibition leads to tumor metastasis and poor clinical outcome [14,16]. Downregulation of let-7/miR-98 family members in various types of cancer results in increased expression of LIN28 [18], which is an important regulator of developmental timing [19] and is associated with tumorigenesis, metastasis, and poor clinical outcome [20]. The mammalian homologs of LIN28, LIN28A and LIN28B, bind to the terminal loops of the precursors of let-7/miR-98 family miRNAs [21]. LIN28 activation promotes cancer cell proliferation and metastasis by suppressing the expression of let-7 [20,22,23], a tumor-suppressive miRNA whose downregulation in NSCLC patients is correlated with poor prognosis [24,25]. 

Curcumin is a polyphenol found in turmeric that has anti-oxidant and anti-cancer effects in various malignancies [26,27,28]. Curcumin was shown to inhibit of zeste homolog 2 (EZH2)-induced tumor cell proliferation, EMT, and metastasis in breast and lung cancers [29,30]. We previously showed that curcumin inhibits the invasion and metastasis of lung cancer cells by suppressing the expression of nuclear factor (NF)-κB and matrix metalloproteinases (MMPs) [31]. Curcumin inhibits lung cancer cell migration and metastasis by inducing the expression of miRNAs such as those in the let-7 family [32]. Difluorinated curcumin is a novel analog of curcumin that has antioxidant properties and induces the expression of tumor-suppressive miRNAs including let-7, miR-26a, miR-101, miR-146a, and miR-200c [33,34]. However, it is unknown whether curcumin regulates miR-98 and LIN28A in NSCLC metastasis. 

To address this issue, the present study investigated the anti-cancer mechanism of curcumin and its effect on miR-98 expression. Our results indicate that curcumin treatment induces miR-98 expression and suppresses lung cancer invasion and migration by decreasing the levels of MMP2 and MMP9. These findings provide insight into molecular mechanisms underlying the anti-cancer effects of curcumin.

## 2. Results

### 2.1. Curcumin Suppresses MMP2/9 Levels and Lung Cancer Growth In Vivo

**Figure 1:**
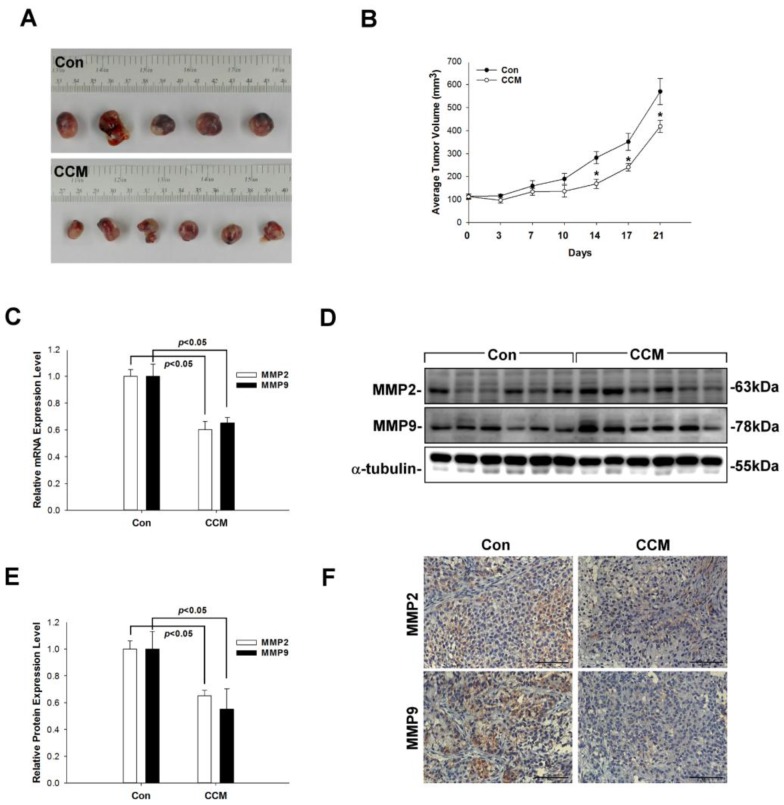
Curcumin inhibits tumor growth and MMP2/9 expression in a xenograft model. (**A**,**B**) Severe combined immunodeficiency mice were inoculated in the right flank with A549 cells. Tumor volume was measured every 3 days with slide calipers starting from day 7, and a growth curve was plotted. Tumors were weighed at the end of the experiment; each plot shows mean ± SEM of six mice per group. * *p* < 0.05 curcumin (CCM) vs. control (Con). (**C**–**F**) *MMP2* and *MMP9* mRNA expression was determined by qRT-PCR (**C**), and protein expression was evaluated by western blotting (**D**,**E**) and confirmed by immunohistochemistry (**F**).

To investigate the anti-tumor effects of curcumin in vivo, A549 cells were subcutaneously transplanted into the flanks of severe combined immunodeficiency mice and mice body weight and tumor growth was monitored. Mice were euthanized 21 days post-implantation, the body weight (Con: 24.96 ± 1.68 g; CCM: 23.08 ± 1.05 g), tumor weight (Con: 1.19 ± 0.11 g; CCM: 0.84 ± 0.08 g) and tumor volume were measured (Con: 570.0 ± 56.1 mm^3^; CCM: 418.3 ± 26.5 mm^3^). Curcumin administration suppressed tumor growth relative to control mice treated with dimethylsulfoxide (DMSO) (Figure 1A). This trend continued through the end of the study period on day 21 (*p* < 0.05; Figure 1B). To clarify the inhibitory role of curcumin in the in vitro growth progression of the lung cancer cell line A549, we evaluated MMP2/9 expression. MMP2/9 were downregulated in curcumin-treated mice as compared to controls, as determined by quantitative real-time (qRT) PCR and western blotting (*p* < 0.05; Figure 1C–E); this result was supported by immunohistochemical analysis of MMP2/9 expression (Figure 1F). These data indicate that curcumin inhibits MMP2/9 expression and tumor growth in a xenograft model of lung cancer.

### 2.2. Curcumin Induces miR-98 Upregulation and LIN28A Downregulation In Vivo

**Figure 2:**
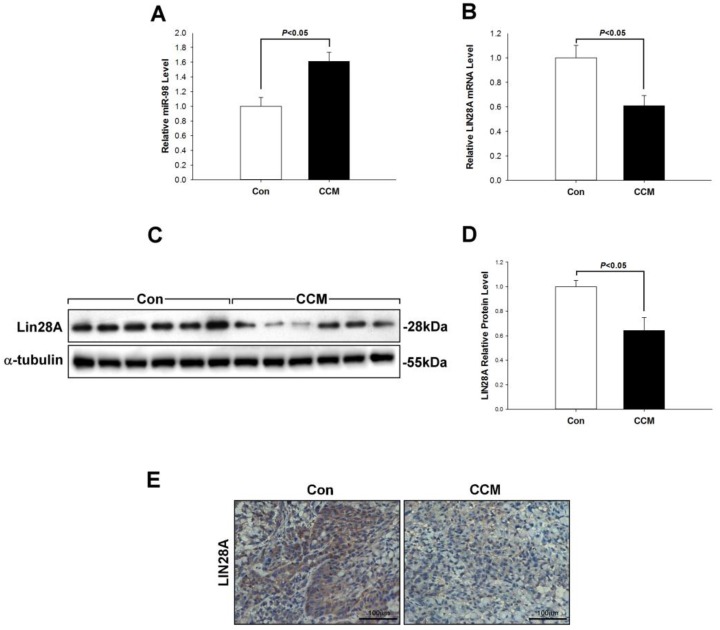
Curcumin induces miR-98 and inhibits LIN28A expression in tumors. (**A**–**E**) MiR-98 level was increased in mice treated with curcumin relative to control mice (**A**), whereas LIN28A mRNA (**B**) and protein (**C**–**E**) levels showed the opposite trend. Protein levels detected by western blotting were semi-quantitatively analyzed by densitometry. Data are shown as mean ± SEM and are representative of three independent experiments.

To evaluate the anti-metastatic mechanism of curcumin in lung cancer, we examined the expression of miR-98 and LIN28A. The qRT-PCR analysis revealed that miR-98 level was markedly increased in the curcumin group relative to controls (*p* < 0.05, Figure 2A). In contrast, LIN28A expression was decreased, as determined by qRT-PCR and western blotting (*p* < 0.05, Figure 2B–D). The latter observation was confirmed by immunohistochemistry (Figure 2F).

### 2.3. Curcumin Inhibits Lung Cancer Cell Migration and Invasion via Upregulation of miR-98

**Figure 3:**
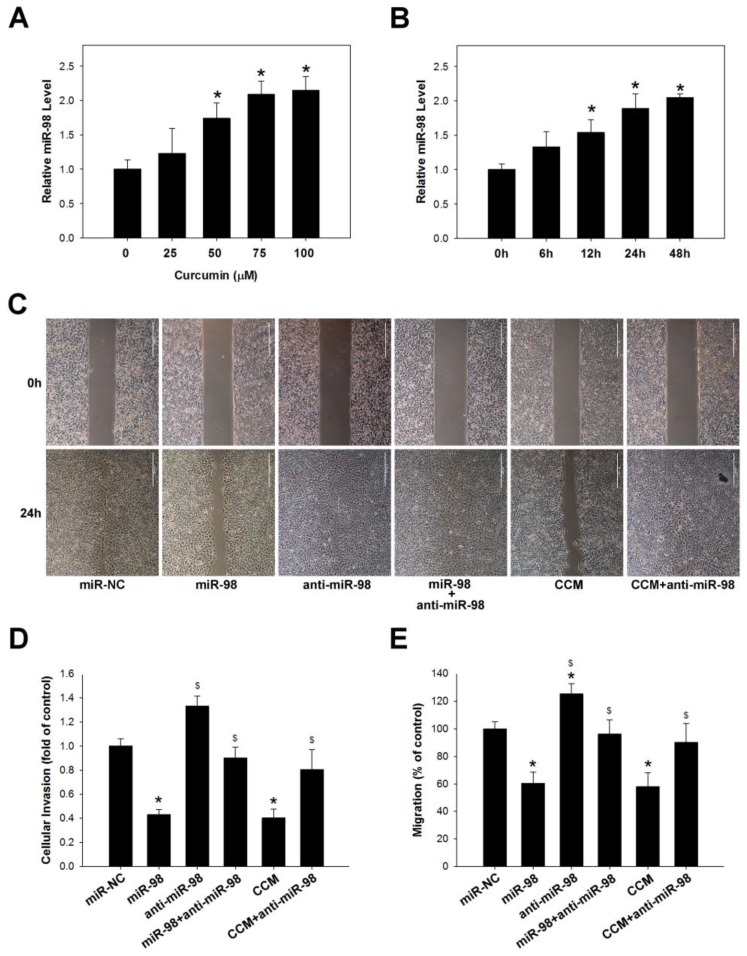
Curcumin inhibits lung cancer cell migration and invasion by regulating miR-98 expression. (**A**,**B**) Curcumin increased miR-98 level in A549 cells in a dose- and time-dependent manner (100 μm curcumin), as determined by qRT-PCR. * *p* < 0.05 vs. untreated control group. (**C**–**E**) MiR-98 (15 nM) overexpression and curcumin (100 μm) treatment inhibits A549 cell migration (**C**,**D**) and invasion (**E**), effects that are abolished by miR-98 inhibitor (anti-miR-98; 15 nM). Data represent mean ± SEM of three independent experiments. * *p* < 0.05 vs. miR-NC group. ^$^ *p* < 0.05 vs. miR-98 group.

Curcumin was previously shown to suppress MMPs expression in lung cancer cells as well as NSCLC metastasis [31]. It was also found to exert anti-cancer effects by regulating miRNA expression [35]. We therefore explored whether curcumin modulates miR-98 expression in lung cancer cells treated with various concentrations of curcumin (25–100 μM) for 6–48 h. The qRT-PCR analysis revealed that miR-98 expression was induced in A549 in a dose- and time-dependent manner (*p* < 0.05; Figure 3A,B). We also examined whether curcumin affects lung cancer cell metastasis via modulation of miR-98 by migration and invasion assays. A549 cells transfected with miR-98 mimic (15 nM) showed reduced cell migration and invasion as compared to those transfected with a negative control construct; however, this effect was abrogated by transfection of miR-98 inhibitor (15 nM) (*p* < 0.05; Figure 3C–E). These results suggest that curcumin prevents lung cancer metastasis by inhibiting cancer cell migration and invasion via modulation of miR-98.

### 2.4. LIN28A Is a Downstream Target of miR-98 in Human Lung Cancer Cells

**Figure 4:**
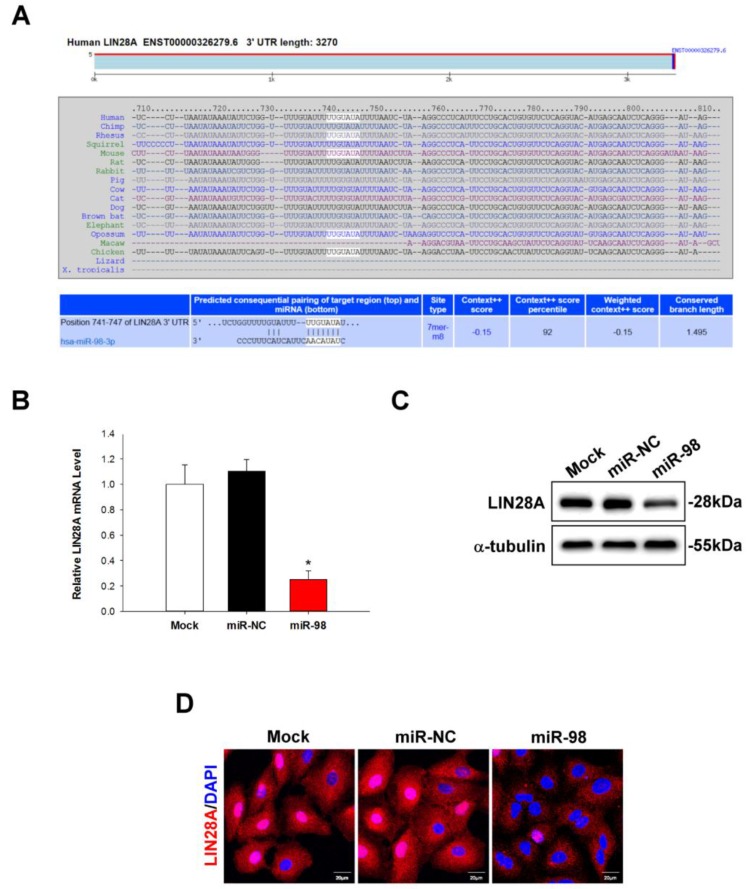
LIN28A is a downstream targets of miR-98 in human lung cancer cells. (**A**) Putative miR-98 binding site in the 3′-UTR of the *LIN28A* gene. (**B**–**D**) LIN28A mRNA expression was evaluated by qRT-PCR, and protein levels were determined by western blotting and immunocytochemistry. Data represent the mean ± SEM of three independent experiments. * *p* < 0.05 vs. untreated control group.

To investigate the relationship between miR-98 and LIN28A in lung cancer, we used TargetScan software to identify downstream targets of miR-98. We found a miR-98-3p binding site (NM_024674) at position 741–747 in the 3′ untranslated region (3′-UTR) of *LIN28A* (Figure 4A). To determine whether LIN28A is a miR-98 target, A549 cells were transfected with miR-98 mimic for 48 h and LIN28A mRNA and protein expression levels were evaluated by qRT-PCR and western blotting, respectively. MiR-98 overexpression inhibited *LIN28A* mRNA (*p* < 0.01; Figure 4B) and protein (Figure 4C) expression relative to mock- or negative control-transfected cells; this was confirmed by immunohistochemistry (Figure 4D). These results indicate that miR-98 directly inhibits LIN28A expression by binding to its 3′-UTR.

### 2.5. Curcumin Inhibits LIN28A-Mediated MMP2/9 Expression and Lung Cancer Metastasis

**Figure 5:**
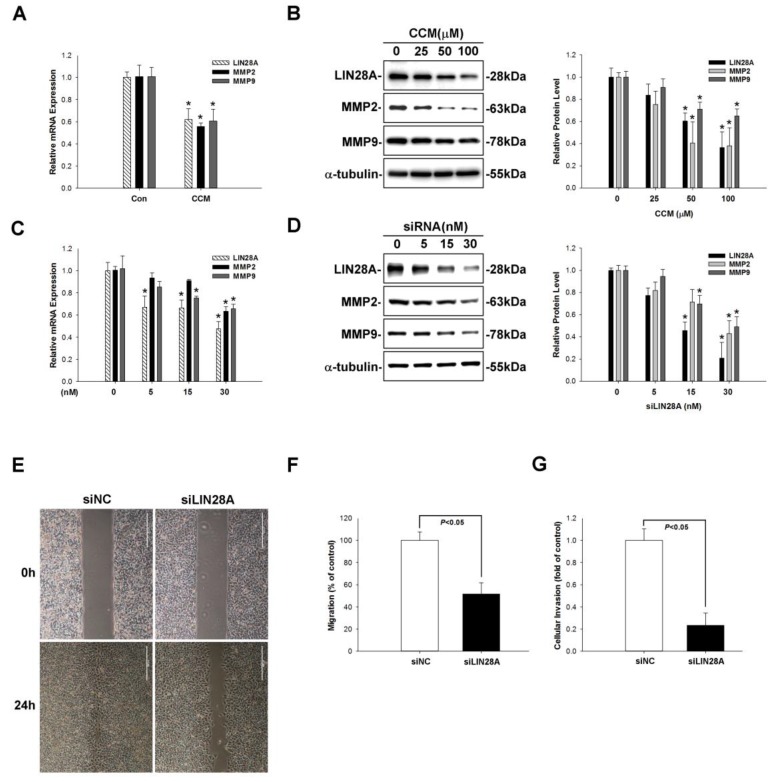
Curcumin reduces LIN28A-induced MMP2/9 expression and blocks cancer metastasis. (**A**) Curcumin (100 μm) treatment decreased LIN28A expression. (**B**) Various curcumin (25, 50, 100 µm) treatment reduced LIN28A, MMP2/9 protein expression. (**C**,**D**) LIN28A silencing decreased LIN28A expression and MMP2/9 mRNA and protein expression in A549 cells, as determined by qRT-PCR and western blotting, respectively. LIN28A (30nM) knockdown significantly suppressed A549 cell migration (**E,F**) and invasion (**G**). Data represent mean ± SEM of three independent experiments. * *p* < 0.05 vs. untreated control group.

Previous studies have reported that LIN28A overexpression induces cancer cell migration and invasion [36,37]. MMPs play a central role in these processes [38]. We therefore examined the effect of curcumin on the expression of LIN28A and MMPs. A549 cells were treated with curcumin (25–100 μM for 24 h) and LIN28A and MMP2/MMP9 levels were evaluated by qRT-PCR and western blotting. Curcumin treatment decreased LIN28A and MMP2/9 mRNA and protein expression (*p* < 0.05; Figure 5A,B). To determine whether LIN28A regulates the expression of MMP2/9, A549 cells were transfected with LIN28A or negative control short interfering (si) RNA (5–30 nM) for 48 h, and LIN28A and MMP2/9 expression was evaluated. *LIN28A* silencing suppressed MMP2/9 levels (*p* < 0.05; Figure 5C,D). To determine whether the downregulation of MMP2/9 caused by loss of LIN28A influenced lung cancer cell metastasis, we carried out migration and invasion assays in A549 cells transfected with LIN28A siRNA. *LIN28A* knockdown suppressed both migration and invasion (Figure 5E–G), indicating that curcumin inhibits MMP2/9 expression via suppression of LIN28A in lung cancer cells.

## 3. Discussion

The results of this study demonstrate for the first time that curcumin-induced miR-98 expression inhibits MMP2/9 expression via modulation of LIN28A and consequently, human lung cancer cell growth in vitro and in vivo. These findings suggest that curcumin can be an effective therapeutic agent for blocking lung cancer progression.

LIN28A overexpression is associated with tumor progression in colon cancer [39]. LIN28A was found to be upregulated in breast cancer and enhanced tumor growth and progression via regulation of c-myc signaling; LIN28A knockdown decreased tumor malignancy [40]. However, there is little information available regarding the role of LIN28A in lung cancer. In this study, we showed that LIN28A expression was associated with MMP2/9 expression in A549 lung carcinoma cells; LIN28A silencing decreased MMP2/9 levels and metastasis. Thus, LIN28A is a marker for tumor cell invasiveness as well as a potential therapeutic target. LIN28A also regulates the stemness of cancer stem cells and is thought to have an oncogenic role in gastrointestinal cancer and leukemia [41]. LIN28 is a specific, post-transcriptional inhibitor of let-7 biogenesis; let-7b overexpression suppresses MMP9 expression in melanoma cells [42]. Consistent with these observations, we found that loss of LIN28A reduced MMP9 expression.

MiRNA dysregulation is a major contributor to cancer biology [43]. MiR-98 is located in an intron of the *HECT*, *UBA*, and *WWE domain-containing 1* gene on the short arm of the X chromosome [3], and acts as a tumor suppressor by targeting EZH2 and LIN28 in various cancers [44,45]. EZH2 is an oncogene that is upregulated in human epithelial-type cancers such as NSCLC [46,47] and whose expression is inhibited by let-7/miR-98 family members [9], along with that of c-Myc [48], high mobility group AT-hook 2 [49], and LIN28 [17]. MiR-98 was observed to be downregulated in various cancer cell lines, and its overexpression inhibited hepatocellular carcinoma (HCC) cell proliferation, migration, and invasion in vitro [50] via suppression of NF-κB p65 nuclear translocation and MMP9 [51] and Sal-like protein 4 expression in HCC and NSCLC cells [15,52]. In accordance with these reports, we found that miR-98 overexpression caused LIN28A downregulation and blocked lung cancer cell migration/invasion and metastasis. These findings indicate that miR-98 functions as a tumor suppressor in lung cancer.

Curcumin has anti-cancer effects and is a potential therapeutic agent for the treatment of various malignancies. Curcumin alters miRNA expression via regulation of p53, Akt, B cell lymphoma-2 (Bcl-2), NOTCH1, and EZH2 signaling pathways [30,53]. However, the precise mechanism underlying the effects of curcumin on miR-98 and LIN28A expression in lung cancer remain unknown. Curcumin has been shown to block cancer metastasis via induction of let-7 and suppression of EZH2, NF-κB, and LIN28 expression [54], and we recently demonstrated that curcumin inhibits A549 cell migration and invasion via negative regulation of NF-κB/MMPs signaling [31]. In this study, curcumin was confirmed to block lung cancer progression via modulation of miR-98, LIN28A, and MMP2/9 levels. These findings provide new insight into the molecular mechanisms of lung cancer progression as well as evidence that curcumin can be an effective therapeutic agent in NSCLC treatment.

Our study has certain limitations. First, this study is not able to differentiate between proMMP2/9 and activated MMP2/9, and cannot justify about the activation status of these proteases. Most antibodies can recognize proMMP2/9 and activated MMP2/9, but there are antibodies that only recognize the prodomain and will only allow us to discriminate between pro/active MMP9. Second, we only performed western blot analysis on cell extracts and not culture supernatant. During the metastatic cascade, changes in cell-cell and cell-matrix adhesion are of paramount importance [55]. Although MMPs might also have intracellular functions, they are predominantly known as secreted enzymes (with functions in ECM remodeling). Additionally, care should be taken when specifying bands as pro/activated MMP2/9 since intracellular MMP2/9 goes through several maturation steps involving differences in molecular weight due to glycosylation and not activation [56]. Finally, an important question remains whether the effects seen on MMP2/9 are a direct effect of miR-98/LIN28A. Xu et al. found that LIN28A overexpression resulted in upregulation of MMP2/9, while LIN28A knockdown downregulated the expression of MMP/9 in pancreatic cancer cell line PANC1 cells, indicating that LIN28A might be critical for invasion of cancer cells [57]. By contrast, this can also be an indirect effect mediated by other signaling pathways affecting MMP2/9 expression. For example, based on NF-kB signaling or Wnt-signaling which has been previously described for curcumin and miR-98 [31,58]. It is still a highly relevant question since many signal pathways are involved in the regulation of MMP2/9 expression. Therefore, the results of this study need to be carefully applied to human subjects. More studies to evaluate how the signal regulations of MMP activity may be related to reduce cancer metastasis are warranted for further clarity.

## 4. Materials and Methods

### 4.1. Culture of A549 Cells

Lung adenocarcinoma cell culture was performed as described previously [31]. A549 cells (ATCC number CCL-185^TM^) were cultured in F12K medium (Thermo Fisher Scientific, Waltham, MA, USA) supplemented with 100 pg/mL of streptomycin (Sigma, Saint Louis, MO, USA), 100 units/mL of penicillin (Sigma) and 5% fetal bovine serum (Invitrogen, Carlsbad, CA, USA), at 37 °C in 95% air-5% CO_2_ condition. Culture medium was changed every 4 days, and cell passages between 4 and 13 times were used for experiments.

### 4.2. Immunohistochemical Staining

Immunohistochemical staining was performed as described previously [31]. Briefly, cells were cultured on glass coverslips, washed with cold PBS, and fixed with 4% paraformaldehyde in PBS at 4 °C for 15 min. After blocking, cells were incubated with the primary antibody against LIN28A (1:250; GeneTex Biotechnology, Irvine, CA , USA), MMP2 (1:400; NeoMarkers, Fremont, CA, USA) and MMP9 (1:400; Santa Cruz Biotechnology, Inc., Santa Cruz, CA, USA) overnight at 4 °C, rinsed with PBS, and incubated with rhodamine-conjugated secondary antibodies for 1 h at 25 °C; cell nuclei were stained with DAPI. After washing with PBS, cells were mounted in Vectashield mounting medium (Vector Laboratories, Burlingame, CA, USA) and examined under a Leica microscope (Wetzlar, Germany). 

### 4.3. Immunocytochemistry

Immunocytochemistry analysis was performed as described previously [59]. Briefly, cells were cultured on glass coverslips, washed with cold PBS, and fixed with 4% paraformaldehyde in PBS at 4 °C for 15 min. After blocking, cells were incubated with the primary antibody against LIN28A (1:100; GeneTex Biotechnology), overnight at 4 °C, rinsed with PBS, and incubated with rhodamine-conjugated secondary antibodies for 1 h at 25 °C; cell nuclei were stained with DAPI. After washing with PBS, cells were mounted in Vectashield mounting medium and examined under a FV1000 confocal laser scanning microscope (Olympus, Center Valley, PA, USA). 

### 4.4. Migration Analysis

The migration assay was performed as described previously [31]. To determine the migration ability of A549 cells, IBIDI*™* Culture Inserts (IBIDI, Martinsried, Germany) were placed into 35-mm culture dishes and 1 × 10^5^ cells/mL were added into the two reservoirs of the same insert. After 24 h, the insert was removed with caution creating a gap of 0.5 mm and cell migration was monitored by bright-field microscopy at specific time points. The cells migrated into the denuded area were photographed and cell-covered areas were measured using the Wimasis WimScratch software. The experiments were performed in triplicate. 

### 4.5. Cell Invasion Assay

Cell invasion was assessed by a modified Matrigel Boyden chamber assay [31] using Bio-Coat Matrigel invasion chambers (BD Biosciences, Bedford, MA, USA) according to the manufacturer’s instructions. Cells (1 × 10^5^ per mL) in serum-free medium were seeded onto Matrigel-coated filters, and 5% FBS was added to the lower chambers as a chemoattractant. After incubation for 24 h, membranes were washed briefly with PBS and the upper side of the membrane was wiped gently with a cotton ball. The cells invaded the lower side of the membrane were removed by Tris-EDTA buffer (10 mM Tris-HCl, pH 8, 0.1 mM EDTA) and counted. 

### 4.6. Quantitative Real-Time PCR

Quantitative real-time PCR was performed as described previously [31]. Total RNA (2 μg) was reverse-transcribed using the SuperScript First-Strand Synthesis System for RT-PCR (Invitrogen) and miRNA was extracted using a miRNA extraction kit (Life Technologies, Carlsbad, CA, USA). The resultant cDNA diluted 1:10 was used as a template to quantify the relative content of mRNA by real-time TaqMan PCR (LightCycler FastStart DNA Master SYBR Green I, Roche, Indianapolis, IN, USA); cDNA diluted 1:5 was used as a standard. The following primers obtained from Integrated DNA Technologies (MDBio, Taipei, Taiwan) were used: LIN28A forward: 5′-CAA AAG GAA AGA GCA TGC AGA A-3′, reverse: 5′-ATG ATC TAG ACC TCC ACA GTT GTA GC-3′; MMP2 forward: 5′-TCC AAC CAC CAC CAC AC-3′, reverse: 5′-AGT CCA AAG AAC TTC TGC AT-3′; MMP9 forward: 5′-TCC AAC CAC CAC CAC AC-3′, reverse: 5′-CGG ACT CAA AGG CAC AGT A-3′; GAPDH forward: 5′-AGC CAC ATC GCT CAG ACA-3′, reverse: 5′-GCC CAA TAC GAC CAA ATC C-3′. U6 (P01183321) primer and miRNA-98 (P01495350) were obtained from Life Science Technology (Gaithersburg, MD, USA). Relative mRNA expression was calculated by normalizing target mRNA levels to those of house-keeping genes (GAPDH and U6) and compared by the CT (ΔΔCT) method.

### 4.7. Cell Transfection with miRNA-98 Mimic and Inhibitor

A549 cells were transfected with miR-98-3p mimic (MC24466; Life Science Technologies) or miR-98-3p inhibitor (MH24466; Life Science Technologies) using the Lipofectamine RNAiMAX kit (Thermo Fisher Scientific). Transfection mixtures containing 0.25 mL of Lipofectamine 2000 (Life Science Technologies), 25 mL of Opti-MEM (Life Science Technologies) and 15 nM miR-98 mimic were incubated at room temperature for 10 min, and added to A549 cells seeded in 6-well plates in 10% FBS-containing medium. Cells were harvested after 48 h and analyzed for miRNA expression using the miRNA extraction kit and quantitative RT-PCR. 

### 4.8. Western Blotting 

Cells were washed with PBS and lysed in protein lysis buffer (BioRad, Hercules, CA, USA) containing protease inhibitors. Cytoplasmic protein extracts were separated in 10% SDS-PAGE gels and transferred to polyvinylidene difluoride membranes for 1 h at room temperature. The membranes were incubated overnight at 4 °C with primary antibodies against LIN28A (1:500; NeoMarkers), MMP2 (1:500; Santa Cruz Biotechnology; mice) and (1:500; GeneTex Biotechnology; human), MMP9 (1:500; Santa Cruz Biotechnology; mice) and (1:500; GeneTex Biotechnology; human); and α-tubulin (1:2000; Sigma). After incubation with appropriate horseradish peroxidase-labeled secondary antibodies for 1 h at room temperature, protein bands were detected using ECL-Plus reagent (EMD Millipore, Billerica, MA, USA) and Biomax MR Film (Kodak, Rochester, NY, USA), and relative protein expression was quantified by densitometry using the ImageQuant 5.2 software (Healthcare Bio-Sciences, Philadelphia, PA, USA).

### 4.9. Animal Study

Twelve male 6–8 week-old SCID mice were purchased from BioLasco Company (Taipei, Taiwan) and quarantined for a week. Animals were housed in a special pathogen-free room with a 12-h light/12-h dark cycle and 40–70% humidity at 19–25 °C. All animals had access to standard rodent diet and water *ad libitum*. Animals (*n* = 6/group) were subcutaneously inoculated (in the flanks) with 0.1 mL PBS containing 1 × 10^7^ A549 cells. Curcumin dissolved in DMSO at 50 mg/kg was injected i.p. 5 day/week/3weeks. DMSO was injected i.p. as placebo control. Mice were weighed every third day to evaluate drug toxicity. Tumor volume was measured every third day to follow the tumor growth. Tumor volume was calculated as T (mm^3^) = length (mm) × width (mm^2^)/2. The protocol for animal study was reviewed and approved by DCB Institutional Animal Care and Use Committee (Approval No. 103034). This experiment was repeated twice.

### 4.10. Statistical Analyses 

The data are presented as the mean ± standard error of mean (SEM) and analyzed by ANOVA and then by Dunnetts’ test. Statistical analysis was performed using SigmaStat version 3.5 (Systat Software Inc., Chicago, IL, USA), and a P value less than 0.05 was considered statistically significant. 

## 5. Conclusions

In this study, we found that curcumin stimulates the expression of miR-98, which in turn negatively regulates LIN28A-induced lung cancer invasion and migration may through inhibition MMP2 and MMP9. These findings clarify the mechanisms of action of curcumin as a potential chemotherapeutic agent for the treatment of lung cancer.
